# Systematic reviews on interventions for COVID-19 have rarely graded the certainty of the evidence

**DOI:** 10.1590/1516-3180.2021.0107.27052021

**Published:** 2021-08-09

**Authors:** Ana Luiza Cabrera Martimbianco, Rafael Leite Pacheco, Carolina de Oliveira Cruz Latorraca, Raphael Einsfeld Simões Ferreira, Rachel Riera

**Affiliations:** I PhD. Professor, Centro Universitário São Camilo, São Paulo (SP), Brazil; and Professor, Universidade Metropolitana de Santos (UNIMES), Santos (SP), Brazil.; II MSc. Professor, Centro Universitário São Camilo, São Paulo (SP), Brazil; and Researcher, Escola Paulista de Medicina, Universidade Federal de São Paulo (EPM-UNIFESP), São Paulo (SP), Brazil.; III MSc. Researcher, Escola Paulista de Medicina, Universidade Federal de São Paulo (EPM-UNIFESP), São Paulo (SP), Brazil.; IV PhD. Professor, Centro Universitário São Camilo, São Paulo (SP), Brazil.; V PhD. Adjunct Professor, Discipline of Evidence-Based Health, Escola Paulista de Medicina, Universidade Federal de São Paulo (EPM-UNIFESP), São Paulo (SP), Brazil; and Coordinator, Health Technology Assessment Center, Hospital Sírio-Libanês, São Paulo (SP), Brazil.

**Keywords:** COVID-19, Systematic review [publication type], Evidence-based practice, GRADE approach, Evidence-based medicine, Public health, Methodology, Certainty of evidence, Strength of recommendations, Evidence-based healthcare

## Abstract

**BACKGROUND:**

Numerous systematic reviews on coronavirus disease-19 (COVID-19) treatment have been developed to provide syntheses of the large volume of primary studies. However, the methodological quality of most of these reviews is questionable and the results provided may therefore present bias.

**OBJECTIVE:**

To investigate how many systematic reviews on the therapeutic or preventive options for COVID-19 assessed the certainty of the evidence through the Grading of Recommendations Assessment, Development and Evaluation (GRADE) approach.

**METHODS:**

We conducted a sensitive search in MEDLINE (via PubMed) and included all systematic reviews that assessed any intervention for COVID-19. The systematic reviews included were examined to identify any planned and/or actual assessment using the GRADE approach (or absence thereof) regarding the certainty of the evidence.

**RESULTS:**

We included 177 systematic reviews and found that only 37 (21%; 37/177) assessed and reported the certainty of the evidence using the GRADE approach. This number reduced to 27 (16.2%; 27/167) when Cochrane reviews (n = 10), in which an evaluation using GRADE is mandatory, were excluded.

**CONCLUSION:**

Most of the systematic reviews on interventions relating to COVID-19 omitted assessment of the certainty of the evidence. This is a critical methodological omission that must not be overlooked in further research, so as to improve the impact and usefulness of syntheses relating to COVID-19.

## INTRODUCTION

Since the beginning of the coronavirus disease 2019 (COVID-19) pandemic, large numbers of studies have been published in an attempt to find an effective treatment for this disease. Consequently, many systematic reviews have been developed on this topic, to provide syntheses of the large volume of primary studies. Healthcare professionals and policymakers commonly use systematic reviews to formulate recommendations and make practical decisions.^1^

However, the methodological quality of most of these systematic reviews is questionable. Hence, the results provided through these reviews may present bias.

Assessing the certainty of the evidence is an indispensable step in a systematic review. This is especially true within the current context, in which information is often misleading yet has been widely disseminated, both by scientific journals and by the traditional media. Thus, efforts need to be made by the authors of syntheses of the evidence on a given topic, to ensure that the degree of certainty that can be placed on the estimates of effect and clinical recommendations can be established.^1^^,^^2^

The Grading of Recommendations Assessment, Development and Evaluation (GRADE) is a transparent approach to rating the certainty of the body of evidence in systematic reviews and other forms of synthesis, as a guide to making decisions.^3^ This approach should be beneficial during the COVID-19 pandemic. It is entirely possible to perform GRADE assessments, even (or even more so) within an emergency context.^1^^,^^4^

## OBJECTIVE

We carried out a critical appraisal study with the aim of investigating how many systematic reviews that have been published in relation to therapeutic or preventive options for COVID-19 made assessments of the certainty of the evidence through the GRADE approach.

## METHODS

We conducted a sensitive search in MEDLINE (via PubMed) on January 20, 2021, using the MeSH term “Coronavirus” and its synonyms combined with the PubMed clinical queries filter for systematic reviews (Annex 1). Two authors independently screened all titles and abstracts through the Rayyan platform,^5^ in order to include systematic reviews that assessed any intervention for COVID-19.

## RESULTS

The systematic reviews included were analyzed in full text, to identify whether there was any planned and/or actual assessment of the certainty of the evidence using the GRADE approach (or absence thereof). The search strategy found 1,075 references, and 177 fulfilled the inclusion criteria. Of these, only 37 reviews (21%; 37/177) assessed the certainty of the evidence using the GRADE approach. This number reduced to 27 (16.2%; 27/167) when Cochrane reviews (n = 10), in which an evaluation using GRADE is mandatory, were excluded.

## CONCLUSION

This result highlights the fact that most of the systematic reviews on interventions conducted in relation to COVID-19 omitted assessment of the certainty of the evidence. This is a critical methodological omission that must not be overlooked in further research, so as to improve the impact and usefulness of syntheses relating to COVID-19.

## Annex 1

**Table:** Search strategy.

Database	Search strategy
	#1 “Coronavirus”[Mesh] OR “Covid-19” OR (COVID) OR (Coronavirus) OR (SARS-CoV-2) OR (Coronaviruses) OR (Deltacoronavirus) OR (Deltacoronaviruses) OR “Munia coronavirus HKU13” OR (Coronavirus HKU15) OR (Coronavirus, Rabbit) OR (Rabbit Coronavirus) OR (Coronaviruses, Rabbit) OR (Rabbit Coronaviruses) OR “Bulbul coronavirus HKU11” OR “Thrush coronavirus HKU12”
MEDLINE (via PubMed)	#2 (((systematic review[ti] OR systematic literature review[ti] OR systematic scoping review[ti] OR systematic narrative review[ti] OR systematic qualitative review[ti] OR systematic evidence review[ti] OR systematic quantitative review[ti] OR systematic meta-review[ti] OR systematic critical review[ti] OR systematic mixed studies review[ti] OR systematic mapping review[ti] OR systematic cochrane review[ti] OR systematic search and review[ti] OR systematic integrative review[ti]) NOT comment[pt] NOT (protocol[ti] OR protocols[ti])) NOT MEDLINE [subset]) OR (Cochrane Database Syst Rev[ta] AND review[pt]) OR systematic review[pt] #3 #1 AND #2
